# Cytogenetics data in adult men involved in the recycling of electronic wastes

**DOI:** 10.1016/j.dib.2018.02.051

**Published:** 2018-02-23

**Authors:** Yanan Du, Yan Wang, Liqing Du, Chang Xu, Kaihua Ji, Jinhan Wang, Qiang Liu

**Affiliations:** Institute of Radiation Medicine, Chinese Academy of Medical Sciences & Peking Union Medical College, Tianjin Key Laboratory of Molecular Nuclear Medicine, Tianjin, China

## Abstract

In this data article, 146 villagers (exposed group) were randomly selected from the workers who involved in the e-wastes recycling directly as a daily job in Tianjin. Control group, including 121 villagers, came from another town without e-waste disposal sites. Chromosomal aberrations (CA) and cytokinesis blocking micronucleus (CBMN) were performed to detect the cytogenetic effect for each subject. DNA damage was detected using comet assay; the DNA percentage in the comet tail (TDNA%), tail moment (TM), and Olive tail moment (OTM) were recorded to describe DNA damage to lymphocytes and spermatozoa. Routine semen analysis, spermatozoa motility and morphology were analyzed. The RT^2^Profiler PCR array was used to measure levels of expression of 84 genes related to quality of DNA. It showed significant relationships between CA, CBMN, DNA damage and exposure time in exposure subjects. The alteration of sperm motility rate, abnormality rate and total sperm counts had association with exposure time and age.

**Specifications table**

**Table:** 

Subject area	Environment and health
More specific subject area	Environmental pollution, cytogenetic alteration
Type of data	Table and figure
How data was acquired	CA and CBMN were acquired using ZEISS MetaSystems (Germany). DNA damage was detected by comet assay.
Data format	Analyzed
Experimental factors	Semen and blood were sampled from the two groups in our lab.
Experimental features	Lymphocytes were cultured in RPMI 1640 medium for CA and CBMN assay. Spermatozoa or lymphocytes were suspended in PBS for comet assay.
Data source location	Institute of Radiation Medicine, Chinese Academy of Medical Sciences & Peking Union Medical College
Data accessibility	All the data are in this data article.

**Value of the data**.

The data were helpful to understand the positive associations between both CA and CBMN and the duration of working with e-wastes.

When stratified for age, for each of the age sub-groups, a statistically significant difference was observed between the group exposed to e-waste and the reference group.

Semen quality was worse in the workers who recycled e-wastes than that of reference subjects.

## 1 Data

**Fig. 1:**
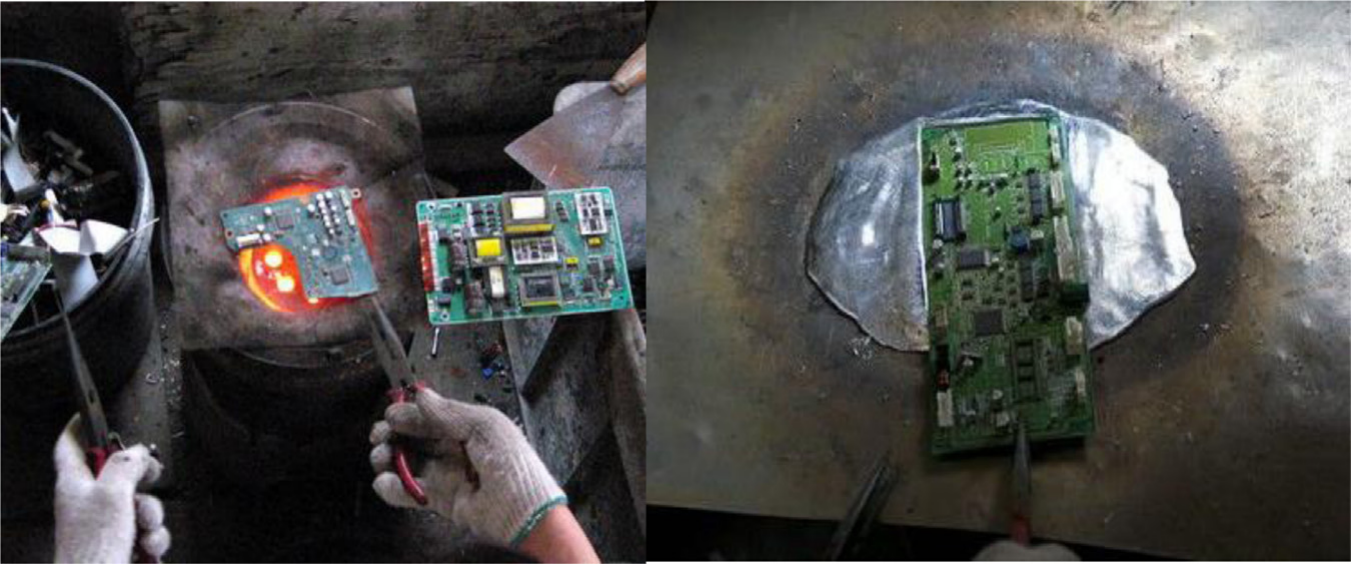
Recycling of e-wastes without any protection. Heat the circuit boards to get metals.

A largest electronic waste disposal centers in northern China had been found recently years. Components of e-wastes such as electronic circuit boards or microchips were illegally burned or heated for reclaimable materials (Fig. 1).

**Fig. 2:**
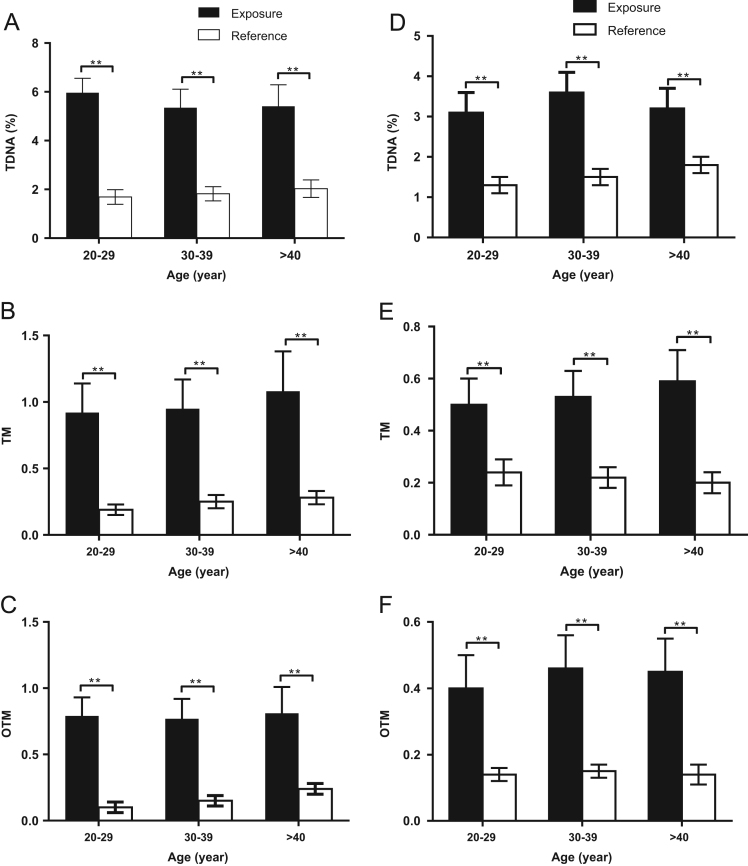
DNA damage detected by comet assay in lymphocytes and spermatozoa for different age sub-groups. The group of workers recycling e-wastes and reference group were both divided into three sub-groups by age (20–29, 30–39 and >40 years old). A-C: TDNA%, TM and OTM in lymphocytes between the group of workers recycling e-wastes and reference group for the sub-groups divided by age. D-F: TDNA%, TM and OTM in spermatozoa between the group of workers recycling e-wastes and reference group for the sub-groups divided by age. **: *P* < 0.01. Two way ANOVA was also used to test the interactions between age and DNA damage in lymphocytes and spermatozoa, respectively. (lymphocytes: *F* = 2.13, *P* = 0.15; spermatozoa: *F* = 1.67, *P* = 0.21).

The exposure and reference group were both divided by age into three sub-groups (20–29, 30–39 and >40 years old). For each age sub-group, significant differences were found between exposure and reference groups (Fig. 2A-F). No significant difference was observed among age-groups in either the exposure or reference group (Fig. 2A-F).

**Fig. 3:**
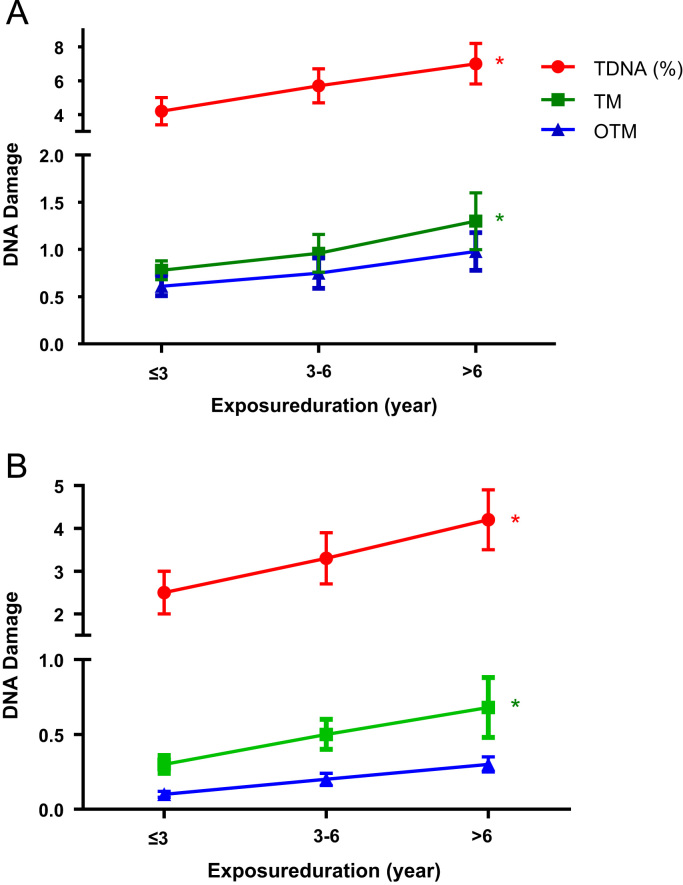
Relationship between exposure duration and DNA damage of lymphocytes (A) and spermatozoa (B) in the exposed group. It showed significant relationship between TDNA%, TM and exposure duration for not only lymphocytes but also spermatozoa. *: *P* < 0.05.

The exposure group was stratified into three sub-groups according to their exposure time (≤ 3, 3–6 and > 6-year groups). The statistical significant relationships between DNA damage (TDNA%, TM) and duration of exposure for DNA damage were found in both lymphocytes and spermatozoa (Fig. 3A and B).

**Fig. 4:**
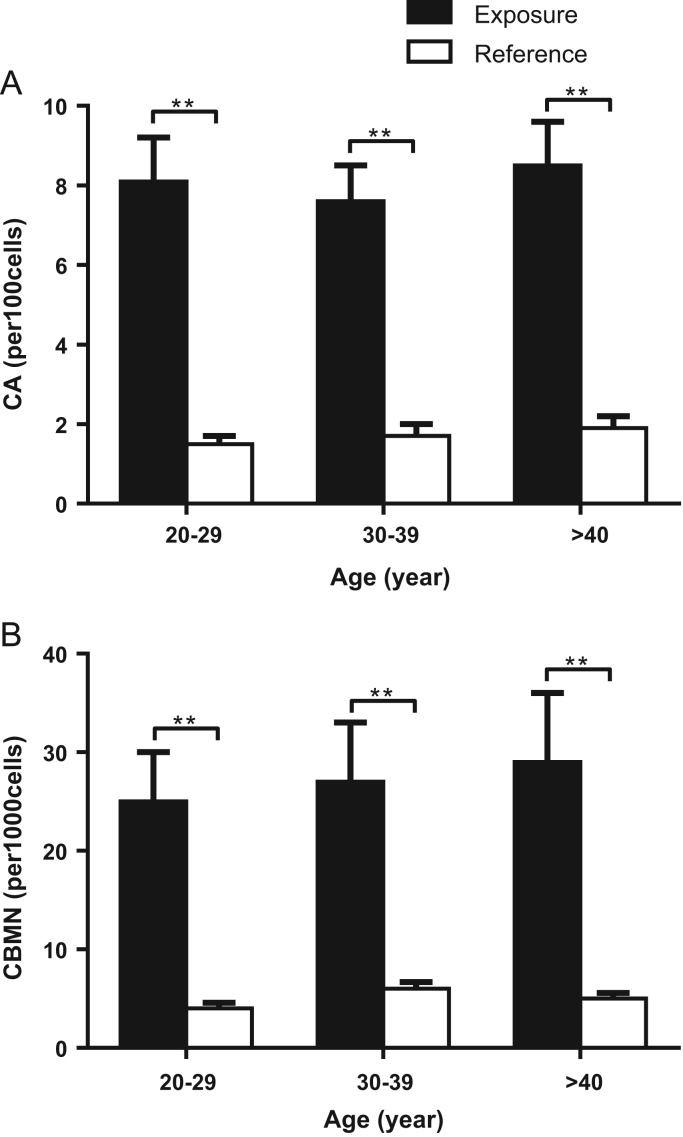
CA (A) and CBMN (B) in lymphocytes of workers recycling e-wastes and in reference group for different age sub-groups. A-B: CA and CBMN in lymphocytes between the group of workers recycling e-wastes and reference group for the sub-groups divided by age. There is no difference of CA and CBMN among the age sub-groups. **: *P* < 0.01. Two way ANOVA was also used to test the interactions between age and CA, CBMN in lymphocytes, respectively. (CA: *F* = 2.03, *P* = 0.18; CBMN: *F* = 1.07, *P* = 0.39).

For each of the sub-groups divided by age, there was significantly higher of CA and CBMN in the e-waste workers compared to the reference group (Fig. 4A and B). No significant difference was found among sub-groups in either the exposure or reference group (Fig. 4A and B).

**Fig. 5:**
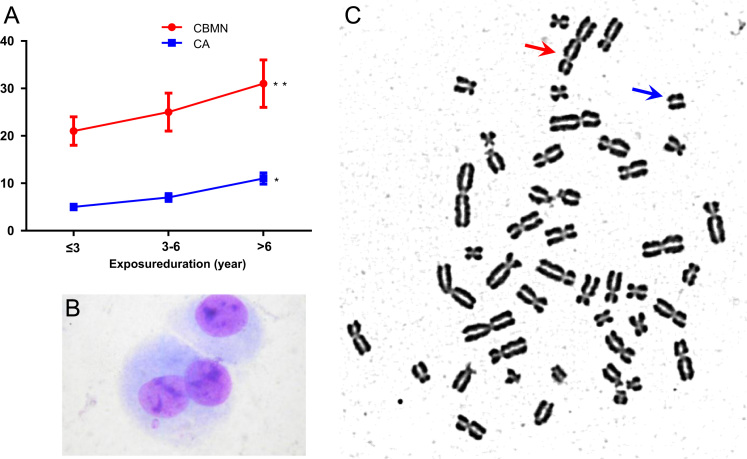
Relationship between exposure duration and frequency of CA and CBMN in lymphocytes of exposed subjects. A: It shows significant relationships between CA, CBMN and exposure duration in lymphocytes. B: One micronucleus in a binucleated lymphocyte of the group of workers recycling e-wastes. C: A metaphase lymphocyte of the group of workers recycling e-wastes. The red arrow directs a dicentric chromosome; the blue arrow directs an acentric fragment. **: *P* < 0.01, *: *P* < 0.05.

Statistically significant was found between CA, CBMN and exposure time (Fig. 5A). A classical micronucleus in a binucleated lymphocyte, a dicentric chromosome and an acentric fragment are shown in Fig. 5B and C.

**Fig. 6:**
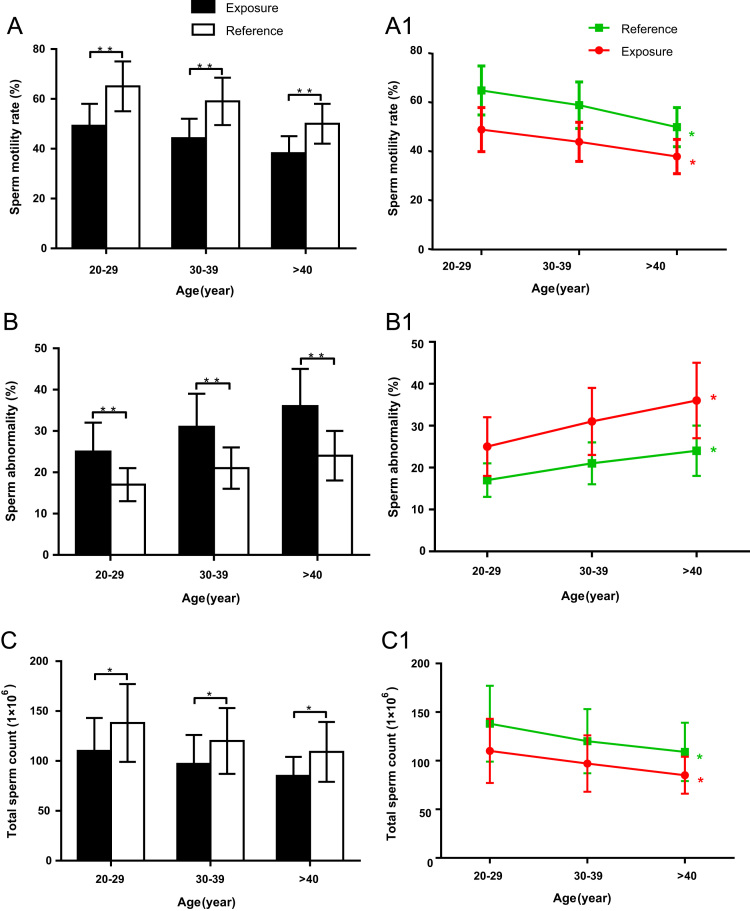
Sperm motility rate, abnormality rate and total sperm counts for different age sub-groups. A-C: Sperm motility rate, abnormality rate and total sperm counts between the group of workers recycling e-wastes and reference group for the sub-groups divided by age. There is significant difference of sperm motility rate, abnormality rate and total sperm counts between the group of workers recycling e-wastes and reference group in the age sub-groups, respectively. A1-C1: Line chart showed the change of sperm motility rate, abnormality rate and total sperm counts along with age. There is significant difference of sperm motility rate, abnormality rate and total sperm counts among the age sub-groups in exposure or reference group respectively. **: *P* < 0.01, *: *P* < 0.05. Two way ANOVA was also used to test the interactions between age and sperm motility rate, abnormality rate and total sperm counts, respectively. (sperm motility rate: *F* = 3.24, *P* = 0.07; abnormality rate: *F* = 3.13, *P* = 0.08; total counts: *F* = 2.89, *P* = 0.12).

Sperm motility rate, abnormality rate and total sperm counts were analyzed in the three sub-groups divided by age for exposure and reference groups. For the same age sub-groups, significant difference was found between exposure and reference group (Fig. 6A, B and C). The sperm parameters above also showed significant difference among different sub-groups in exposure or reference group respectively (Fig. 6A1, B1 and C1).

**Fig. 7:**
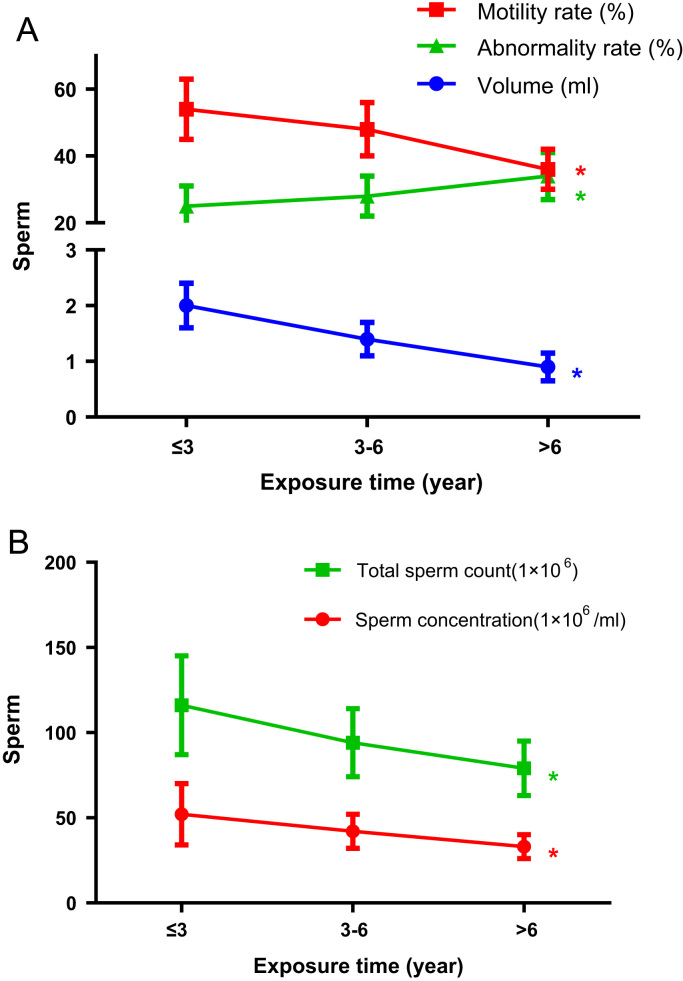
Relationship among semen parameters and exposure duration. A: Sperm motility rate and semen volume decreased but sperm abnormality rate increased significantly with e-wastes exposure duration. B: Sperm concentration and total sperm count both decreased significantly with exposure duration. *: *P* < 0.05.

Relationship between semen alteration and exposure time of e-wastes was analyzed in exposure group. For the three sub-groups divided by exposure time (≤ 3, 3–6 and >6years groups), semen parameters were analyzed for every two sub-groups by Wilcoxon rank-sum test. Sperm motility rate, semen volume, sperm concentration and total sperm count decreased significantly with exposure time, however, sperm abnormality rate increased significantly with e-wastes exposure time (Fig. 7).

RNA of peripheral blood cells was isolated by use of the RNeasy Mini kit (Qiagen, Hilden, Germany) as instructed by the manufacturer. Integrity of RNA was assessed by means of the Bioanalyzer 2100 (Agilent Technologies, Palo Alto, CA). 84 key genes (Table 1) from Human DNA Damage Signaling Pathway were simultaneously assayed by use of the RT2Profiler PCR array plate (SuperArray Bioscience Corporation, Frederick, MD) according to the manufacturer's protocol. The detail of gene expression analysis was shown in Ref. [1].

**Table 1:** Gene table of the 84 genes assayed with Human DNA Damage Signaling pathway PCR array RT2Profiler.

**No.**	**Unigene**	**GeneBank**	**Symbol**	**Description**	**Gene Name**
1	Hs.431048	NM_005157	ABL1	C-abl oncogene 1, receptor tyrosine kinase	ABL/JTK7
2	Hs.601206	NM_198889	ANKRD17	Ankyrin repeat domain 17	GTAR/NY-BR-16
3	Hs.73722	NM_080649	APEX1	APEX nuclease (multifunctional DNA repair enzyme) 1	APE/APE-1
4	Hs.367437	NM_000051	ATM	Ataxia telangiectasia mutated	AT1/ATA
5	Hs.271791	NM_001184	ATR	Ataxia telangiectasia and Rad3 related	FRP1/MEC1
6	Hs.533526	NM_000489	ATRX	Alpha thalassemia/mental retardation syndrome X-linked (RAD54 homolog, S. cerevisiae)	ATR2/MRXHF1
7	Hs.194143	NM_007294	BRCA1	Breast cancer 1, early onset	BRCAI/BRCC1
8	Hs.519162	NM_006763	BTG2	BTG family, member 2	PC3/TIS21
9	Hs.292524	NM_001239	CCNH	Cyclin H	CAK/p34
10	Hs.184298	NM_001799	CDK7	Cyclin-dependent kinase 7	CAK1/CDKN7
11	Hs.24529	NM_001274	CHEK1	CHK1 checkpoint homolog (S. pombe)	CHK1
12	Hs.291363	NM_007194	CHEK2	CHK2 checkpoint homolog (S. pombe)	CDS1/CHK2
13	Hs.135471	NM_006384	CIB1	Calcium and integrin binding 1 (calmyrin)	CIB/KIP
14	Hs.249129	NM_001279	CIDEA	Cell death-inducing DFFA-like effector a	CIDE-A
15	Hs.151573	NM_004075	CRY1	Cryptochrome 1 (photolyase-like)	PHLL1
16	Hs.290758	NM_001923	DDB1	Damage-specific DNA binding protein 1, 127 kDa	DDBA/UV-DDB1
17	Hs.505777	NM_004083	DDIT3	DNA-damage-inducible transcript 3	CEBPZ/CHOP
18	Hs.339396	NM_007068	DMC1	DMC1 dosage suppressor of mck1 homolog, meiosis-specific homologous recombination (yeast)	DMC1H/HsLim15
19	Hs.435981	NM_001983	ERCC1	Excision repair cross-complementing rodent repair deficiency, complementation group 1 (includes overlapping antisense sequence)	COFS4/UV20
20	Hs.487294	NM_000400	ERCC2	Excision repair cross-complementing rodent repair deficiency, complementation group 2 (xeroderma pigmentosum D)	COFS2/EM9
21	Hs.498248	NM_130398	EXO1	Exonuclease 1	HEX1/hExoI
22	Hs.591084	NM_004629	FANCG	Fanconi anemia, complementation group G	FAG/XRCC9
23	Hs.409065	NM_004111	FEN1	Flap structure-specific endonuclease 1	FEN-1/MF1
24	Hs.292493	NM_001469	XRCC6	X-ray repair complementing defective repair in Chinese hamster cells 6 (Ku autoantigen, 70 kDa)	CTC75/CTCBF
25	Hs.80409	NM_001924	GADD45A	Growth arrest and DNA-damage-inducible, alpha	DDIT1/GADD45
26	Hs.9701	NM_006705	GADD45G	Growth arrest and DNA-damage-inducible, gamma	CR6/DDIT2
27	Hs.661218	NM_002066	GML	Glycosylphosphatidylinositol anchored molecule like protein	LY6DL
28	Hs.577202	NM_005316	GTF2H1	General transcription factor IIH, polypeptide 1, 62 kDa	BTF2/TFIIH
29	Hs.191356	NM_001515	GTF2H2	General transcription factor IIH, polypeptide 2, 44 kDa	BTF2/BTF2P44
30	Hs.386189	NM_016426	GTSE1	G-2 and S-phase expressed 1	B99
31	Hs.152983	NM_004507	HUS1	HUS1 checkpoint homolog (*S. pombe*)	Hus1
32	Hs.503048	NM_002180	IGHMBP2	Immunoglobulin mu binding protein 2	CATF1/HCSA
33	Hs.17253	NM_054111	IHPK3	Inositol hexaphosphate kinase 3	INSP6K3/IP6K3
34	Hs.61188	NM_033276	XRCC6BP1	XRCC6 binding protein 1	KUB3
35	Hs.1770	NM_000234	LIG1	Ligase I, DNA, ATP-dependent	MGC117397
36	Hs.463978	NM_002758	MAP2K6	Mitogen-activated protein kinase kinase 6	MAPKK6/MEK6
37	Hs.432642	NM_002969	MAPK12	Mitogen-activated protein kinase 12	ERK3/ERK6
38	Hs.35947	NM_003925	MBD4	Methyl-CpG binding domain protein 4	MED1
39	Hs.195364	NM_000249	MLH1	MutL homolog 1, colon cancer, nonpolyposis type 2 (*E. coli*)	COCA2/FCC2
40	Hs.436650	NM_014381	MLH3	MutL homolog 3 (*E. coli*)	HNPCC7
41	Hs.509523	NM_002431	MNAT1	Menage a trois homolog 1, cyclin H assembly factor (*Xenopus laevis*)	MAT1/RNF66
42	Hs.459596	NM_002434	MPG	N-methylpurine-DNA glycosylase	AAG/APNG
43	Hs.192649	NM_005590	MRE11A	MRE11 meiotic recombination 11 homolog A (*S. cerevisiae*)	ATLD/HNGS1
44	Hs.597656	NM_000251	MSH2	MutS homolog 2, colon cancer, nonpolyposis type 1 (*E. coli*)	COCA1/FCC1
45	Hs.280987	NM_002439	MSH3	MutS homolog 3 (*E. coli*)	DUP/MRP1
46	Hs.271353	NM_012222	MUTYH	MutY homolog (*E. coli*)	MYH
47	Hs.396494	NM_018177	N4BP2	Nedd4 binding protein 2	B3BP
48	Hs.492208	NM_002485	NBN	Nibrin	AT-V1/AT-V2
49	Hs.66196	NM_002528	NTHL1	Nth endonuclease III-like 1 (*E. coli*)	NTH1/OCTS3
50	Hs.380271	NM_002542	OGG1	8-oxoguanine DNA glycosylase	HMMH/HOGG1
51	Hs.20930	NM_020418	PCBP4	Poly(rC) binding protein 4	LIP4/MCG10
52	Hs.147433	NM_182649	PCNA	Proliferating cell nuclear antigen	MGC8367
53	Hs.424932	NM_004208	AIFM1	Apoptosis-inducing factor, mitochondrion-associated, 1	AIF/PDCD8
54	Hs.111749	NM_000534	PMS1	PMS1 postmeiotic segregation increased 1 (*S. cerevisiae*)	DKFZp781M0253/HNPCC3
55	Hs.632637	NM_000535	PMS2	PMS2 postmeiotic segregation increased 2 (*S. cerevisiae*)	HNPCC4/PMS2CL
56	Hs.225784	NM_005395	PMS2L3	Postmeiotic segregation increased 2-like 3	PMS2L9/PMS5
57	Hs.78016	NM_007254	PNKP	Polynucleotide kinase 3'-phosphatase	PNK
58	Hs.631593	NM_014330	PPP1R15A	Protein phosphatase 1, regulatory (inhibitor) subunit 15A	GADD34
59	Hs.700597	NM_006904	PRKDC	Protein kinase, DNA-activated, catalytic polypeptide	DNAPK/DNPK1
60	Hs.531879	NM_002853	RAD1	RAD1 homolog (*S. pombe*)	HRAD1/REC1
61	Hs.16184	NM_002873	RAD17	RAD17 homolog (*S. pombe*)	CCYC/HRAD17
62	Hs.375684	NM_020165	RAD18	RAD18 homolog (*S. cerevisiae*)	RNF73
63	Hs.81848	NM_006265	RAD21	RAD21 homolog (*S. pombe*)	HR21/HRAD21
64	Hs.655835	NM_005732	RAD50	RAD50 homolog (*S. cerevisiae*)	RAD50-2/hRad50
65	Hs.631709	NM_002875	RAD51	RAD51 homolog (RecA homolog, E. coli) (S. cerevisiae)	BRCC5/HRAD51
66	Hs.172587	NM_133509	RAD51L1	RAD51-like 1 (*S. cerevisiae*)	R51H2/RAD51B
67	Hs.655354	NM_004584	RAD9A	RAD9 homolog A (*S. pombe*)	RAD9
68	Hs.546282	NM_002894	RBBP8	Retinoblastoma binding protein 8	CTIP/RIM
69	Hs.443077	NM_016316	REV1	REV1 homolog (*S. cerevisiae*)	REV1L
70	Hs.461925	NM_002945	RPA1	Replication protein A1, 70 kDa	HSSB/REPA1
71	Hs.408846	NM_022367	SEMA4A	Sema domain, immunoglobulin domain (Ig), transmembrane domain (TM) and short cytoplasmic domain, (semaphorin) 4A	CORD10/RP35
72	Hs.591336	NM_014454	SESN1	Sestrin 1	PA26/SEST1
73	Hs.211602	NM_006306	SMC1A	Structural maintenance of chromosomes 1A	CDLS2/DKFZp686L19178
74	Hs.81424	NM_003352	SUMO1	SMT3 suppressor of mif two 3 homolog 1 (*S. cerevisiae*)	DAP-1/GMP1
75	Hs.654481	NM_000546	TP53	Tumor protein p53	LFS1/TRP53
76	Hs.697294	NM_005427	TP73	Tumor protein p73	P73
77	Hs.694840	NM_016381	TREX1	Three prime repair exonuclease 1	AGS1/AGS5
78	Hs.191334	NM_003362	UNG	Uracil-DNA glycosylase	DGU/DKFZp781L1143
79	Hs.654364	NM_000380	XPA	Xeroderma pigmentosum, complementation group A	XP1/XPAC
80	Hs.475538	NM_004628	XPC	Xeroderma pigmentosum, complementation group C	XP3/XPCC
81	Hs.98493	NM_006297	XRCC1	X-ray repair complementing defective repair in Chinese hamster cells 1	RCC
82	Hs.647093	NM_005431	XRCC2	X-ray repair complementing defective repair in Chinese hamster cells 2	DKFZp781P0919
83	Hs.592325	NM_005432	XRCC3	X-ray repair complementing defective repair in Chinese hamster cells 3	XRCC3
84	Hs.444451	NM_016653	ZAK	Sterile alpha motif and leucine zipper containing kinase AZK	AZK/MLK7

## 2 Experimental design, materials and methods

### 2.1 Instruments and reagents

Agarose gels with normal and low melting points were purchased from the Biowest Company (Miami, FL, USA). Tris–HCl, DMSO, NaHCO3, formaldehyde (A.P.), trypan blue and TritonX-100 were purchased from Sigma (St. Louis, MO, USA). The electrophoresis apparatus was purchased from BIO-RAD (Hercules, CA, USA), and the Nikon90i fluorescence microscope was purchased from NIKON (Tokyo, Japan). The comet slides were purchased from Trevigen. Inc. (Gaithersburg, MD, USA).

### 2.2 Routine semen analysis

The procedure of routine semen analysis was performed according to the standard methods in the WHO manual [2]. Briefly, the semen samples were examined immediately after liquefaction or within one hour of ejaculation. All the semen samples were ensured to be homogeneous by mixing thoroughly. A fixed volume of 10 μl semen was delivered onto a clean glass slide and covered with a coverslip. Scanning the slide and estimating the number of spermatozoa per 400× magnification field gives an approximate sperm concentration in 106/ml. This estimate is used to decide the dilution (1:5, 1:10, 1:20, 1:50) for determining the sperm concentration by hemocytometry. The spermatozoa concentration was determined using the hemocytometer method on two separate preparations of the semen sample. The diluted semen sample was dropped onto the hemocytometer and covered with a coverslip, and was placed in a humid chamber for about five minutes to prevent drying out. The cells sedimented during this time and were then counted. The count only included complete spermatozoa (heads with tails). Any sperm lying on the line dividing two adjacent squares was counted only if it was on the upper or the left side of the square being assessed.

### 2.3 Alkaline Comet assay

Spermatozoa or lymphocytes were suspended in PBS at a concentration of 1 × 105/ml. The comet assay, also called single cell gel electrophoresis, was performed as previously reported [3]. Briefly, comet slides were coated with 100 μl of 0.75% (w/v) normal-melting-point agarose. Once the first agarose layer was coagulated, a mixture of 75 µl of low-melting-point agarose and 25 µl of spermatozoa suspension was applied as the second layer. The comet slides were immersed in cold lysis buffer (2.5 M NaCl, 0.5 M EDTA, 10 mM Tris HCl pH 10.0 containing 1% Triton X-100, 40 mM dithiothreitol and 100 μg/ml proteinase K) for 2 h at room temperature to remove any DNA-associated proteins. After lysis, double-distilled water was used to rinse away excess salt. All the comet slides were then placed in buffer for 20 min in a horizontal electrophoresis tank that was pre-filled with cold alkaline buffer (1 mM Na2EDTA and 0.3 M NaOH, pH 13.0) to loosen the tight double-helical structure of DNA for electrophoresis. Electrophoresis was then performed at 25 V and 10 mA for 20 min in electrophoresis buffer at room temperature. The slides were then rinsed twice with distilled water and stained with ethidium bromide (2 µg/ml). All of the above procedures were carried out in the dark to avoid additional DNA damage. The comets were viewed using a Nikon 90i fluorescence microscope, and images of 100 comets were collected for each subject using a digital imaging system. Cells that overlapped were not counted. All the comet images were analyzed using Comet Assay Software Project (CASP, Wroclaw University, Poland) [4] and the DNA percentage in the comet tail (TDNA%), the tail moment (TM) and the Olive tail moment (OTM) were recorded to describe the DNA damage to the spermatozoa or lymphocytes.
